# Oleic acid influences the adipogenesis of 3T3-L1 cells via DNA Methylation and may predispose to obesity and obesity-related disorders

**DOI:** 10.1186/s12944-019-1173-6

**Published:** 2019-12-28

**Authors:** Malgorzata Malodobra-Mazur, Aneta Cierzniak, Tadeusz Dobosz

**Affiliations:** Department of Forensic Medicine, Molecular Techniques Unit, Wroclaw Medical University, Sklodowskiej-Curie 52, 50-369 Wroclaw, Poland

**Keywords:** Palmitic acid, Stearic acid, Oleic acid, Adipogenesis, DNA methylation, 3T3-L1, Insulin signaling

## Abstract

**Background:**

Adipogenesis is the process of adipocytes formation from unspecialized progenitor cells called mesenchymal stromal cells. Numerous mechanisms including epigenetic regulation modulate the correct progress of this process. Dietary exposures occurring over a specific period of time might cause long-lasting and even permanent changes in gene expression regulated by epigenetic mechanisms. For that reason, we investigated the adipogenesis of 3 T3-L1 cells with the excess of saturated and monounsaturated fatty acids and their influence on global and site-specific DNA methylation in these cells.

**Materials and methods:**

3T3-L1 cells were cultured in vitro to obtain 100% of confluence, then the adipogenesis was induced by a differentiation cocktail with the addition of the excess of 0.25 mM and 0.5 mM of palmitic (16:0), stearic (18:0) and oleic (18:1n-9) acids. DNA and RNA were extracted at five-time points to assess the adipogenesis process. The phenotype of mature adipocytes (insulin sensitivity, adipokines secretion, fat content) was estimated in fully mature adipocytes. DNA methylation was investigated both during adipogenesis and in mature adipocytes.

**Results:**

Oleic acids stimulated expression of *C/ebpα* and *Pparγ*, which was correlated with lower methylation levels at promoters sites. Furthermore, cells cultured with an excess of oleic acid were characterized by higher lipid accumulation rate, higher leptin, and lower adiponectin secretion. Moreover, in all experimental cells, insulin signaling and glucose utilization were impaired.

**Conclusion:**

Oleic acid affected the methylation of *Pparγ* and *C/ebpα* promoters, what correlated with higher expression. Furthermore, examined free fatty acids influenced the phenotype of mature adipocytes, especially insulin signaling pathway and adipokine secretion.

## Introduction

Adipogenesis is the process of formation of adipocytes from unspecialized progenitor cells known as mesenchymal stromal cells (MSC) [1]. The process of differentiating these cells in vitro requires a pharmacological cocktail containing glucocorticoids (dexamethasone), cAMP stimulating agent (IBMX − 3-Isobutyl-1-methylxanthine), and insulin in order to induce both insulin receptor and insulin-like growth factor expression [2]. Administration of the above-mentioned agents to confluent 3T3-L1 cells results in several mitotic divisions known collectively as mitotic clonal expansion (MCE), followed by inhibition of growth and induction of the expression of transcriptional factors controlling adipogenesis [3]. Adipogenesis begins with the induction of the expression by the differentiation cocktail components of early transcription factors, i.e. *C/ebpβ (*CCAAT/enhancer binding protein, beta) and *C/*ebpδ (CCAAT/enhancer binding protein, delta), directly or indirectly (via *Creb* (cAMP responsive element binding protein) and others). In turn, these early transcription factors induce the expression of late transcription factors, mainly *Pparγ* (peroxisome proliferator activated receptor gamma) and *C/ebpα* (CCAAT/enhancer binding protein, alpha), which induce further expression of adipogenic genes such as *Srebp* (Sterol regulatory element binding protein) and others [1, 3].

The process of adipogenesis is closely controlled by numerous mechanisms, including epigenetic regulation, that modulates its correct progress [4, 5]. Epigenetic regulation is defined as the heritable and reversible modification of gene expression without changes in the DNA sequence maintained over generations [6, 7]. Epigenetic modifications include DNA methylation, positioning of nucleosomes, and histone modifications such as methylation, phosphorylation, and acetylation, as well as a wide range of non-coding RNAs (ncRNAs) [7]. All of these modifications are involved in setting up and maintaining the two forms of chromosomal structure: inactive heterochromatin and transcriptionally active euchromatin. DNA methylation, the most widely studied epigenetic modification, occurs predominantly in the CG-rich genome region known as the CpG island and is reliant on the addition of a methyl group, chiefly in the cytosine residues at the C5 position of CG sites. Hypermethylation of DNA is associated with transcriptional inactivity, whereas hypomethylation of DNA is associated with activation of transcription [6].

Dietary factors may influence the epigenome at two stages: (1) during the fetal and neonatal stages and (2) in the course of lifestyle changes (diet, physical activity) occurring over a long period of time in adult individuals [8]. Dietary exposure (excessive fat, carbohydrates, etc.) over a specific period of time may cause long-lasting and even permanent changes in gene expression regulated by epigenetic mechanisms [8]. A high-fat diet (HFD) has been shown to cause significant changes in DNA methylation in humans [9]. Furthermore, it appears that these changes are subject to slow reversal, indicating that HFD causes long-lasting changes in the DNA methylation profile. Triacylglycerols delivered with the diet are the source of metabolically active free fatty acids (FFA). The influence of FFA on the epigenome has been confirmed by many researchers. Palmitic acids have been shown to influence gene expression and to alter the activity of histone-modifying enzymes (H3K9Ac, H3K4me3, and H3K27me3) in the promoter regions of numerous genes in INS1 β-cells [10]. Palmitic acid has also been implicated in changes in the methylation profile of *TNFα* promoter in 3T3-L1 adipocytes [11]. Oleic acid has been shown to stimulate adipogenesis in hen preadipocytes by increasing the expression of key adipogenic transcription factors such as *C/ebpβ* or *Fabp4* (fatty acid binding protein 4) [12]. Furthermore, the influence of oleic acid on the methylation of mitochondrial DNA (mtDNA) has been reported [13]. Moreover, FFA (as a cocktail of lauric, myristic, linoleic, oleic, and arachidonic acids) have been shown to downregulate miR-143 (well-characterized miRNA involved in adipogenesis) in human adipocytes [14]. The stearic acid also has been shown to stimulate adipogenesis, either by increasing expression of transcription factors or stimulating lipids accumulation [15], however, its influence at the DNA methylation was not displayed so far. Nonetheless, it was selected for study, because is one of the most abundant fatty acid delivered with food.

Epigenetic modification, including DNA methylation, has proven to be an important mediator in the regulation of adipogenesis and thus in the development of obesity-related metabolic disorders. Epigenetic modifications are the best example of gene-environmental interaction. The intake of fat is crucial for health and well-being, however, the excess of fat is related to obesity development and pathogenesis of the obesity-related disorder. We hypothesize that excess fat intake might influence the adipogenesis process through epigenetic mechanisms. Therefore, we investigated the adipogenesis of 3T3-L1 cells with an excess of saturated (palmitic and stearic) and monounsaturated (oleic acid) fatty acids and their influence on global and site-specific DNA methylation rates in these cells and their influence on the phenotype of mature adipocytes.

## Materials and methods

### Cell culture and viability test

3T3-L1 cells (ATCC, CL-173™) were grown in a DMEM medium (Corning), supplemented with 10% fetal calf serum (FCS, Sigma-Aldrich) and containing appropriate antibiotics (penicillin, 50 U/ml; streptomycin, 50 μg/ml, Corning), in a humidified incubator at 37 °C and 5% CO_2_ to achieve 100% confluence.

Prior to the appropriate experiments, the cells were subjected to viability tests using an MTT (3-(4,5-dimethylthiazol-2-yl)-2,5-diphenyltetrazolium bromide, a tetrazole, Sigma-Aldrich) assay in order to assess the maximum concentration of fatty acids (stearic, palmitic, and oleic acids) without an effect on the viability of the cells. First, the cells were incubated for 24 and 48 h in a medium containing fatty acid over a wide range of concentrations (0.1–1 mM) of appropriate fatty acids. For the control cells, BSA was added. Next, 5 mg/ml of MTT solution was added and cells continued to be cultured for 3.5–4 h at 37 °C. In the mitochondria of living cells, yellow MTT was reduced to purple formazan. Following incubation, the medium was removed, wells were washed carefully with PBS, and the purple formazan was dissolved in DMSO (Corning). Absorbance was measured at 590 nm with a reference filter of 620 nm.

Prior to the experiments, the fatty acids (all purchased from Sigma-Aldrich) were dissolved in 0.1 M NaOH (Sigma-Aldrich) and incubated at 70 °C for 30 min. Next, 10% bovine serum albumin (BSA, Sigma-Aldrich), essentially free of fatty acids, was added and further incubated, with constant stirring, for 1 h at 55 °C. Solutions of acids bound with albumin were filtered through a 0.2-μm syringe filter. The final concentration of each fatty acid solution was 25 mM.

### Adipogenesis

Differentiation to mature adipocytes was induced after achieving 100% confluence through the addition of DMEM medium with 10% fetal bovine serum (FBS), containing antibiotics (penicillin, 50 U/ml; streptomycin, 50 μg/ml), 3-isobutyl-1-methylxanthine (115 μg/ml), dexamethasone (390 ng/ml), insulin (10 μg/ml), and the appropriate concentrations (0.25 and 0.50 mM) of fatty acids (palmitic acid 16:0, stearic acid 18:0, oleic acid 18:1n-9). To the control cells 7,5% BSA was added at the concentration equaled the concentration of BSA in fatty acids solution. After 2 days, the medium was changed to DMEM with antibiotics, 10% FBS, insulin (10 μg/ml), and fatty acid (0.25 and 0.50 mM) in experimental cells and BSA in control cells. After 2 more days, the medium was changed to DMEM with antibiotics, 10% FBS, and fatty acids (0.25 and 0.50 mM)) in experimental cells and BSA in control cells. The cells reached maturity after 8 days from the initiation of differentiation.

Five points in time were selected during adipogenesis in order to assess the process: D0 – undifferentiated cell, D0.5–12 h after adipogenesis induction (the MCE), D3 – third day of differentiation, D6 – sixth day of differentiation, and D8 – phenotype of mature adipocytes. At each point, cells were harvested and genetic material was extracted for further experiments. At D8 the medium was collected for ELISA tests.

### DNA and RNA isolation

DNA was isolated from harvested 3T3-L1 cells using the phenol-chloroform-isoamyl method. Stated briefly, adipocytes were washed twice with PBS and suspended in lysis buffer [Tris-HCl (pH 7.5), TEN, 10% SDS] with the addition of 2.5 μl Proteinase (Sigma Aldrich). Next, DNA was extracted using phenol-chloroform-isoamyl alcohol (25/24/1, v/v/v, BioShop) and precipitated with 96% ethanol (StanLab).

Total cellular RNA was isolated using Tri-Reagent (Sigma-Aldrich) followed by RNA purification on a silica membrane using a commercial kit (SV Total RNA Isolation System, Promega).

### Gene expression

Reverse transcription was performed with the use of a High-Capacity cDNA Reverse Transcription Kit (Applied Biosystems), using 200 ng of total RNA. Gene expression was carried out using Real-Time PCR based on a SYBR Green assay. Primers were designed manually to flank two exons of the mRNA (Table 1). The specificity of primers was checked using Primer-BLAST (NCBI); secondary structures were analyzed using OligoAnalyzer (IDT, Inc.). Prior to Real-Time PCR, the efficiency of the primers was analyzed using the standard curve method; their specificity was checked based on the denaturation curve. Only primers with efficiencies of R2 > 0.95 were used in the study. The gene expression was measured using the Fast SYBR Green Master Mix (Thermo Fisher Scientific). The relative gene expression level, normalized to the housekeeping gene (*β-actin*), was calculated using the delta-delta Ct (ΔΔCt) model.

**Table 1:** The sequence of primers used for gene expression study and for bisulfate sequencing study

Gene	Sequence		Location in the gene	Product length [bp]	R^2**^
*Β-actin*	F	CCCAGATCATGTTTGAGACC	2/3^*^	53	0,99
	R	CTGGATGGCTACGTACATG			
*Dnmt1*	F	ATGAGAGGGAGGAGAAGAGAC	9/10^*^	184	0,99
	R	TGCTGCTGGTACTTCAGGTTAG			
*Dnmt3a*	F	CTCAGTGGTGTGTGTGGAGA	2/3^*^	196	0,98
	R	CACTTCCACAGCCTTGCCACT			
*C/ebpα*	F	GACTGGAGTTATGACAAGCTTC	1^*^	107	0,99
	R	TGCACACTGCCATTGCACAAG			
*C/ebpβ*	F	ATCCGGATCAAACGTGGCT	1^*^	107	0,99
	R	AACCCCGCAGGAACATCTTT			
*C/ebpδ*	F	CGAATCGCTAGTTTCTTTGGG	1^*^	75	0,99
	R	ATAGCTTCTCTCGCAGTCCAG			
*Pparγ*	F	ATGTCTCACAATGCCATCAGG	4/5^*^	98	0,99
	R	TCTGGGTTCAGCTGGTCGAT			
*Insr*	F	CCAGGCATCGTGTGAAAATG	6/7^*^	160	0,99
	R	CTCTGTCACATTCTGATAAG			
*Slc2a4*	F	CAGGCATCAATGCTGTTTTC	7/8^*^	109	0,99
	R	GTGAAGACCGTATTGACCAC			
*Lep*	F	TTCACACACGCAGTCGGTAT	1/2^*^	84	0,99
	R	GACAAACTCAGAATGGGGTG			
*Adipoq*	F	AGGAGATGCAGGTCTTCTTG	1/2^*^	137	0,99
	R	CTGAGCGATACACATAAGC			
*Ptpn1*	F	AAAACCTGACTACCAAGGAG	5/6^*^	55	0,99
	R	CATGTGGTGTAGTGGAAATG			
*Pik3r1*	F	GACATCTCAAGGGAAGAAGT	2/3^*^	106	0,99
	R	GTGTAAGAGTGTAATCGCCG			
*Akt1*	F	CATTCAGACTGTGGCAGATG	4/5^*^	145	0,99
	R	AAACTCGTTCATGGTCACAC			
*Fasn*	F	TTGATGATTCAGGGAGTGG	4/5^*^	126	0,99
	R	TGCTGCCATCTGTATTGGTGC			
*Acaca*	F	CTTAGAGAGGGGTCAAGTC	14/15^*^	149	0,99
	R	CTTCCACACACGAGCCATTC			
*Scd1*	F	CCCACATGCTCCAAGAGATC	1/2^*^	127	0,99
	R	TCAGGACGGATGTCTTCTTCC			
*Lpl*	F	TCAACTGGATGGAGGAGGAG	3/4^*^	103	0,99
	R	TTGGTCAGACTTCCTGCTAC			
*Il-6*	F	GATACCACTCCCAACAGACC	2/3^*^	99	0,98
	R	GCACAACTCTTTTCTCATTTC			
*Il-10*	F	TAAGGGTTACTTGGGTTGCC	2/3^*^	144	0,92
	R	CGCATCCTGAGGGTCTTCA			
*C/ebpα*	F	TAGAAGGTGTTGGAGTTGATTAGTG	937–1140 bp	193	
	R	CCTCTAAATCTCCAACCAAAAAAC			
*Pparγ*	F	TTAGAGGAAGGTAAATTTTTGATAG	− 1925 - − 1706 bp	219	
	R	AAACAATCACCCTATCAATCACAC			

^*^ Number of the exon/ exon junction

^**^ primers efficiency assessed based on standard curve method

### Global and site-specific DNA methylation analysis, CpG island prediction

The global methylation of DNA was measured using a MethylFlash Methylated DNA Quantification Kit (Epigentek) according to the manufacturer’s protocol.

The prediction of CpG islands in the promoter region was done using a USCS Genome Browser and MethPrimer software (UCSF). The prediction criteria: CG content > 55% in the region of 500-bp length, ObsCpG/ExpCpG > 0.65. Bisulfite treatment of genomic DNA (500 ng) was performed using an EpiJET Bisulfite Conversion Kit (Thermo Fisher Scientific), according to protocol, in order to convert the unmethylated cytosine to uracil. The amplification of the CpG islands for the appropriate gene was done using a QIAGEN Multiplex PCR Kit (QIAGEN). The primers for PCR were selected based on data obtained during the prediction of the CpG islands in MethPrimer software (UCSF). The sequence of primers used for CpG island amplification is presented in Table 1. Prior to the experiments, the temperature condition of the PCR was determined for each CpG island. The amplification results were checked using agarose-gel electrophoresis. The amplified CpG islands were sequenced using the Sanger method in an outsourced laboratory. The sequencing results were analyzed using QUMA (QUantification Tool for Methylation Analysis, Riken) [16].

### Glucose uptake

Measurements of glucose uptake by experimental and control cells were performed using a Glucose Uptake Assay Kit (Abcam). Stated briefly, 3T3-L1 cells were divided into mature adipocytes with or without an excess of fatty acids on a 6-well culture dish. On the day of full maturation, adipocytes were plated on a 96-well plate at a density of 3000 cells per well. Next, the cells were starved in a serum-free adipocyte medium overnight. Prior the experiment, the cells were starved of glucose via pre-incubation with a glucose-free medium (KRPH (Krebs-Ringer-Phosphate-Hepes buffer)/2%BSA). Next, cells were stimulated with 1 μM insulin for 20 min, following which 10 mM of 2-deoxyglucose (2DG6P) was added to the cells, with further culturing for 20 min. After washing, cells were subjected to further processing according to the manufacturer’s protocol. The results were measured at OD412 nm in kinetic mode at 37 °C, to reach OD = 1.5–2 in the higher concentration of the standard curve sample.

### Adipokines levels

Levels of adipokines were measured in a medium collected at D8 of differentiation using commercial kits: Mouse Adiponectin ELISA Research Reagent (Boster) for mouse adiponectin, the Mouse Leptin ELISA Kit (Sigma-Aldrich) for mouse leptin and Mouse IL-6 ELISA Kit (Millipore) for IL-6.

### Assessment of the accumulated lipids

The amount of accumulated lipids was measured using Oil Red O stain (Sigma-Aldrich). The cells were incubated with 4% paraformaldehyde (PFA, Sigma-Aldrich) for 10 min at RT. The PFA was discarded; the cells were washed with water; then 60% isopropanol (Sigma-Aldrich) was added to the cells for 5 min and removed. The previously prepared Oil Red O solution was added and incubated 30 min at RT. Following incubation, the cells were washed with water until the water was clear. The accumulated Oil Red O in the cells was extracted with 100% isopropanol; the absorbance was measured at 492 nm.

### Statistical analysis

Statistical analysis was carried out using STATISTICA 13.1 (StatSoft). To compare numerical values between experimental and control cells, one-way and/or multifactor ANOVA was used. To assess the difference between each experimental cell and control cells, a posthoc test (the NIR-Fisher test) was used. Statistical significance was set at *p* < 0.05.

## Results

### Viability test using methylthiazolyldiphenyl-tetrazolium bromide (MTT)

The MTT test showed that the viability of 3T3-L1 cultured with an excess of free fatty acids was unchanged up to a concentration of 0.5 mM for each analyzed fatty acid. In concentrations higher than 0.5 mM, the viability of cells decreased dramatically. Therefore, for further experiments, two concentrations were chosen: 1) 0.5 mM of fatty acid, as the highest concentration that did not affect cell viability, and 2) 0.25 mM, as half of the maximum concentration.

**Fig. 1:**
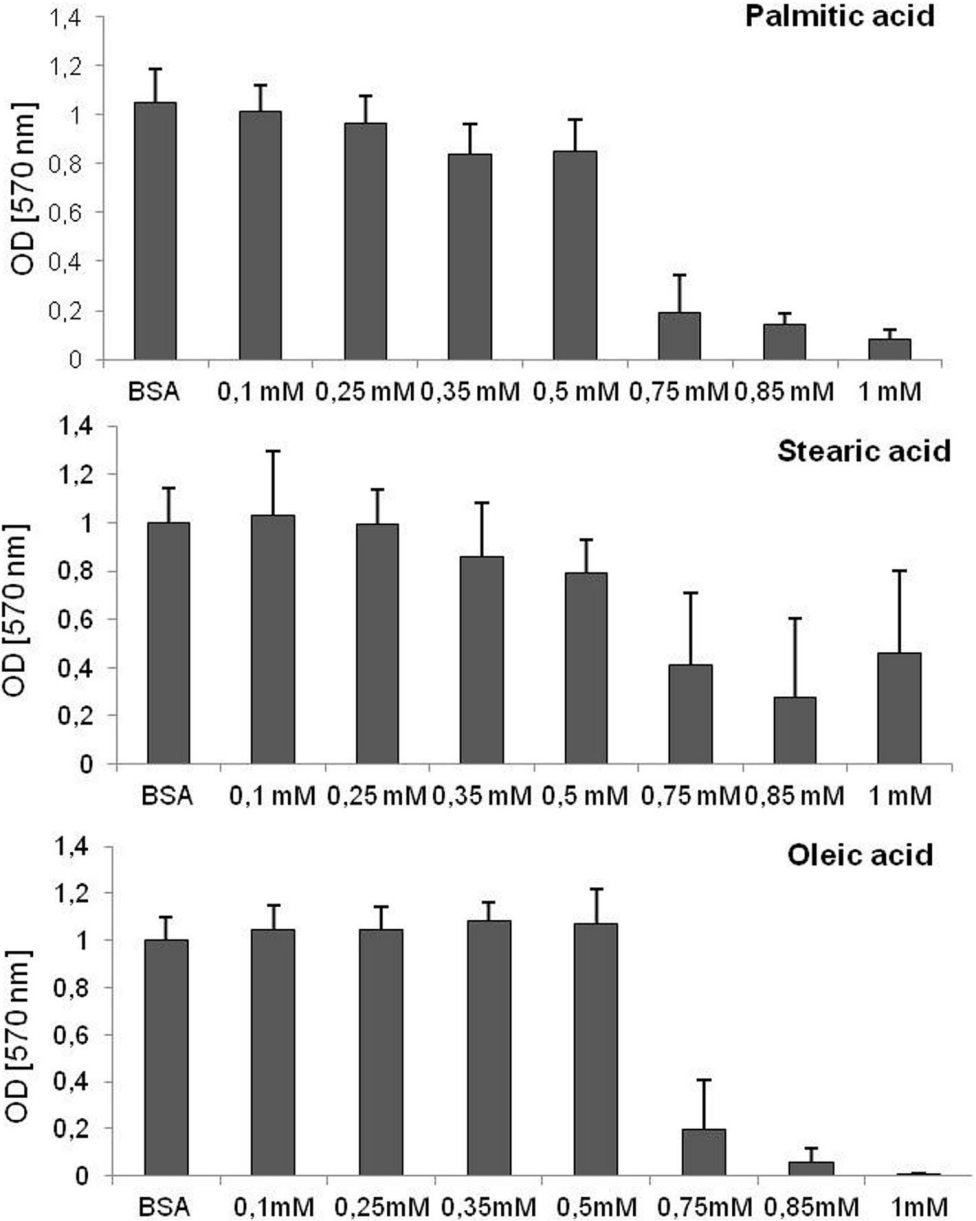
The effect of FFA on cell viability. The viability of 3T3-L1 cells treated with saturated and monounsaturated fatty acids after 48 h of incubation

Graphs depicting viability after 48 h of incubation with a particular fatty acid are presented in Fig. 1.

### Differentiation of 3T3-L1 fibroblasts into mature adipocytes with an excess of palmitic, stearic, and oleic fatty acids (the time-point study)

#### The effect of fatty acids on the adipogenesis of 3 T3-L1 fibroblasts

The differentiation process, carried out for eight days, was monitored during adipogenesis at five points in time: D0 (24 h of incubation with fatty acids, without a differentiation cocktail), D0.5 (12 h after adipogenesis induction by adding a differentiation cocktail), D3, D6, and D8 (mature adipocytes). The following parameters were estimated in order to assess the effect of adipogenesis during the entire process at the established points: 1) morphological changes, 2) changes in the expression of transcription factors (early markers of differentiation: *C/ebpβ* and *C/ebpδ*; late markers of differentiation: *C/ebpα* and *Pparγ*); and 3) accumulation of lipids at the end of differentiation (D8) comparing to undifferentiated cells.

**Fig. 2:**
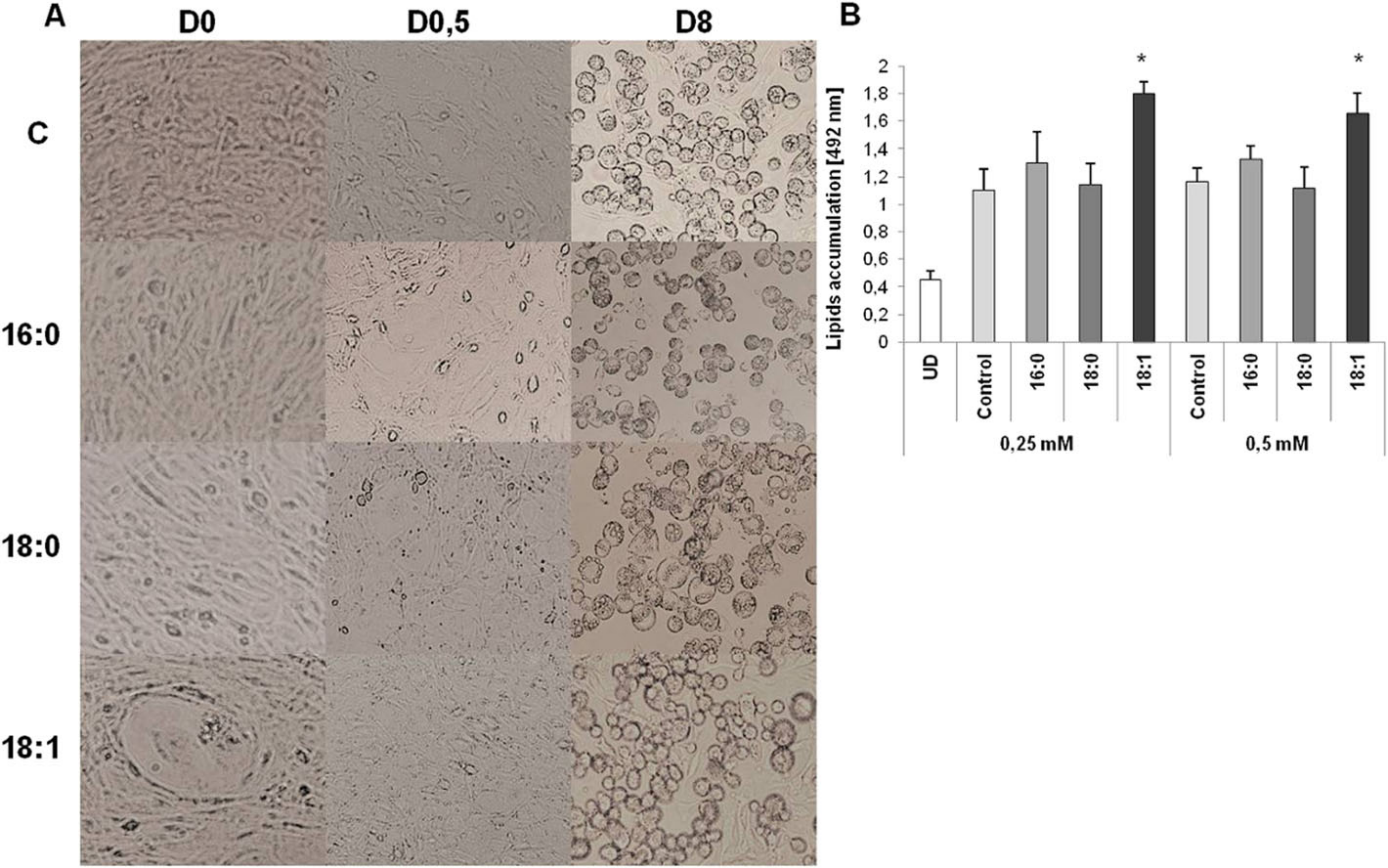
The adipogenesis of 3T3-L1 fibroblasts with the excess of free fatty acids. **a** – morphological changes during adipogenesis (magnification 10x), **b** – total lipids accumulation in mature adipocytes differentiated with the excess of fatty acids at two concentrations [0.25 and 0.5 mM] measured by Oil Red Staining

In cells cultured with oleic acid the lipid droplets appeared much earlier than in control cells and cells cultured with the other two fatty acids (palmitic and stearic acids). At D0 of differentiation in cells treated with oleic acids (for 24 h), the first morphological changes, characterized by the initialization of adipogenesis, began to appear. At D0.5, following adipogenesis initialization in 3T3-L1 treated with oleic acid, changes in structure into round cells with small lipid droplets around the cell were detected (Fig. 2 a). At D8 of differentiation, control cells and cells cultured with an excess of fatty acids [0.25 and 0.5 mM] were characterized by the full phenotype of adipocytes (round cells filled with lipid droplets around cytoplasm, Fig. 2 a). Adipocytes cultured with the addition of oleic acid (at both concentrations) were characterized by higher total lipid content accumulated in mature adipocytes (Fig. 2 b, *p* = 0.0000, for both concentrations), than in control cells and cells cultured with an excess of two other saturated fatty acids. The total lipid content in cells cultured with saturated fats did not statistically differ from that in control cells.

During the differentiation process, the expression of main transcription factors was analyzed in control cells and cells differentiated with fatty acids. There were no differences in the expression rates of early transcription factors driving adipogenesis, i.e., *C/ebpβ* and *C/ebpδ (*data not shown). In control cells and in 3 T3-L1 differentiated with an excess of fatty acids, the expression of *C/ebpβ* reached its maximum at D0.5 following induction of adipogenesis with no differences between experimental cells; this was followed by a decrease in expression during adipogenesis. *C/ebpδ* was expressed at the highest rate at D3 of differentiation, with no differences between experimental and control cells. A similar expression rate was observed at both concentrations of fatty acids.

**Fig. 3:**
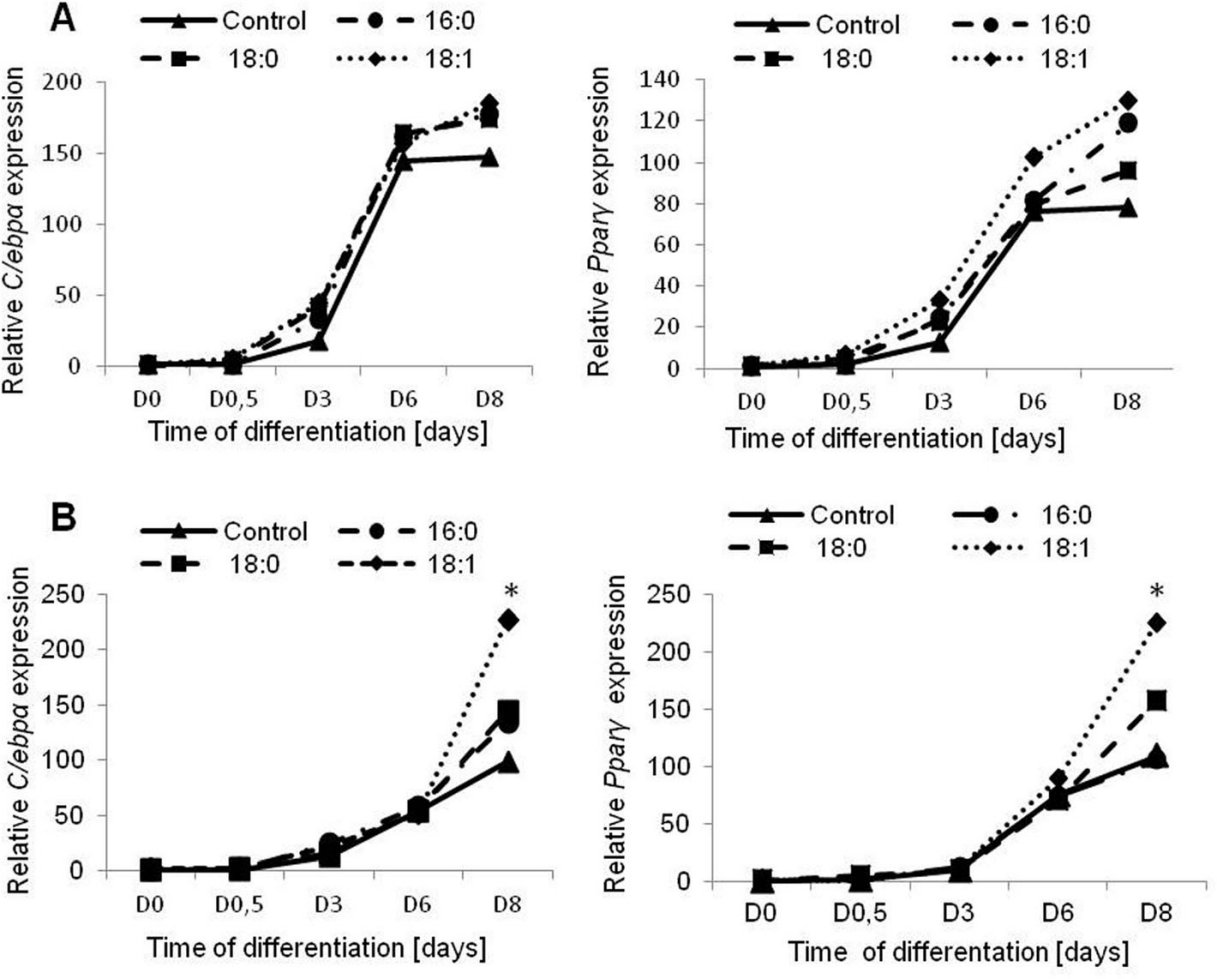
The expression rate of adipogenic transcription factors during 3T3-L1 differentiation. **a** – with the excess of 0.25 mM of free fatty acids, **b** – with the excess of 0.5 mM of free fatty acids. The gene expression presented as relative expression normalized to *β-actin* gene. * *p* < 0.05

The expression rate of late adipogenic transcription factors (*C/ebpα, Pparγ*) began to increase on the third day of differentiation, with the highest level of expression in fully differentiated adipocytes. Expression of both late transcription factors was stimulated by fatty acids, with the greatest influence of oleic acid observed at the end of adipogenesis in comparison to the control cells. A greater increase was observed for oleic acid at the concentration of 0.5 mM (*C/ebpα*, *p* = 0.0222, *Pparγ*, *p* = 0.0551). Palmitic and stearic acids (concentration 0.5 mM) stimulated expression of *C/ebpα* and *Pparγ* moderately, compared to control cells, albeit without statistical significance (Fig. 3).

#### The effect of fatty acids on global DNA methylation during adipogenesis

**Fig. 4:**
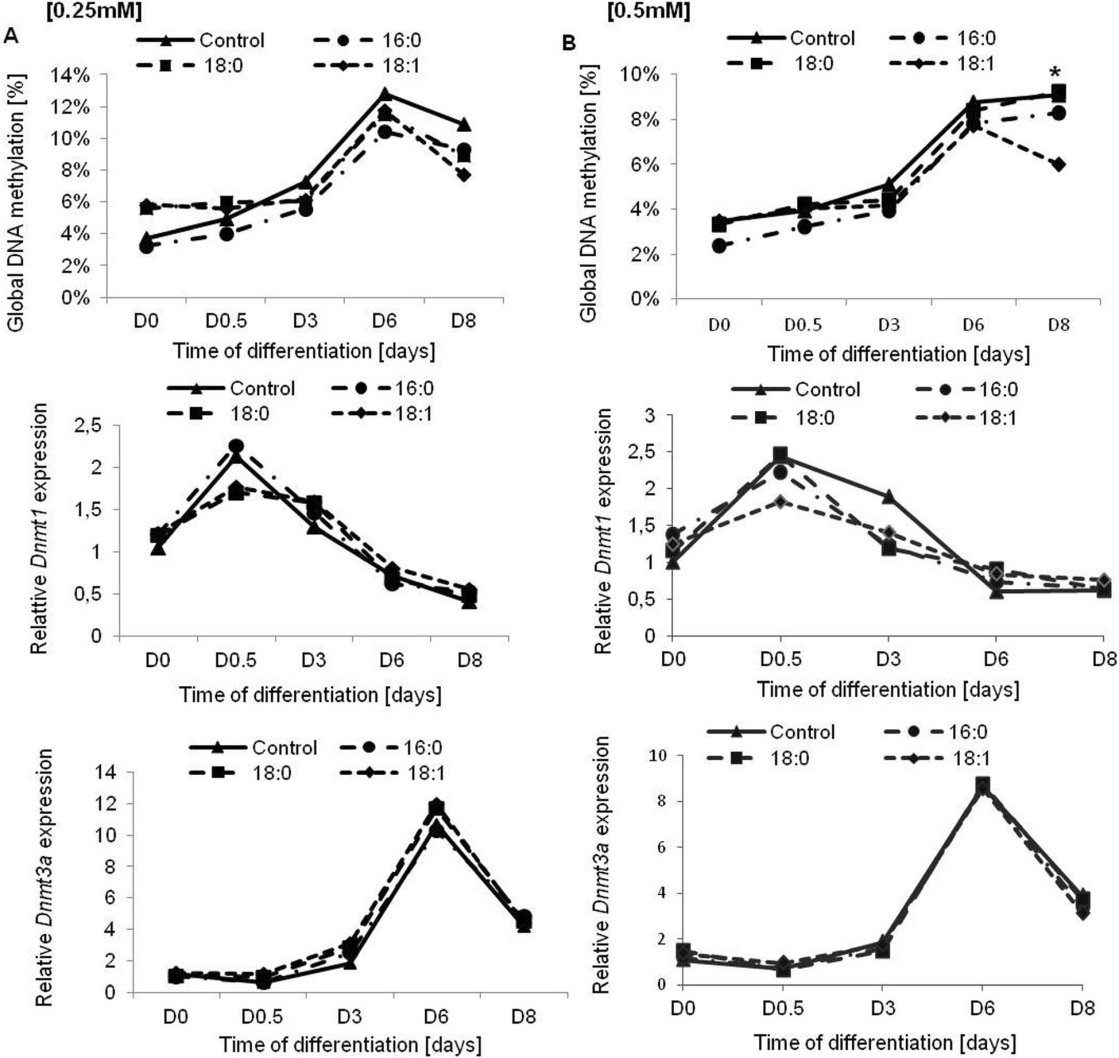
The rate of global DNA methylation (%) and the expression of *DNMTs* during 3T3-L1 differentiation. **a** – the excess of 0.25 mM of free fatty acids, **b** – the excess of 0.5 mM of free fatty acids. The first line corresponds to global DNA methylation, second and third to *Dnmt1* and *Dnmt3a* expression, respectively. The gene expression presented as relative expression normalized to *β-actin* gene. * *p* < 0.05

DNA methylation is one of the well-known factors that influence adipogenesis; therefore, we were interested in learning whether the stimulation of adipogenesis by oleic acids might occur at the level of DNA methylation. Therefore, we measured global DNA methylation during the differentiation of 3T3-L1 cells under experimental conditions. Global DNA methylation increased during adipogenesis in all experimental cells up to D6 of differentiation and declined slightly in fully mature adipocytes. Global DNA methylation was lower in cells treated with fatty acids. The lowest rate of DNA methylation was seen in adipocytes differentiated with oleic acid, but the difference was statistically important only for higher concentrations (0.5 mM, *p* = 0.0486, Fig. 4).

Having noted differential global DNA methylation in cells treated with fatty acids, we next investigated the expression of three main DNA methyltransferases (*Dnmt1*, *Dnmt3a*, and *Dnmt3b*). The expression rate of *Dnmt3b* was very low in 3T3-L1 adipocytes and did not change the expression rate during adipogenesis. The expression of *Dnmt1* increased at D0.5 of differentiation, at the time of mitotic clonal expansion; subsequently, this expression decreased and remained low until full maturation, with no differences between experimental cells. The expression of *Dnmt1* was slightly lower at D0.5 and D3 in cells treated with oleic acid; however, the difference was not statistically significant. *Dnmt3a* increased during adipogenesis in control and experimental cells up to day D6 of differentiation and declined at D8 (similar to the global DNA methylation level) showing no differences in expression rate between experimental and control cells. Similar results were obtained for both fatty acid concentrations [0.25 and 0.5 mM] (Fig. 4).

#### The effect of fatty acids on site-specific DNA methylation during adipogenesis

Next, we became interested in the influence of fatty acids on site-specific DNA methylation profiles, especially in the promoter region of adipogenic transcription factors. Two of the four transcription factors showed different expression rates in experimental and control cells (*C/ebpα* and *Pparγ*). Therefore, the promoters were analyzed using MethPrimer to determined the CpG-rich region within each promoter.

**Fig. 5:**
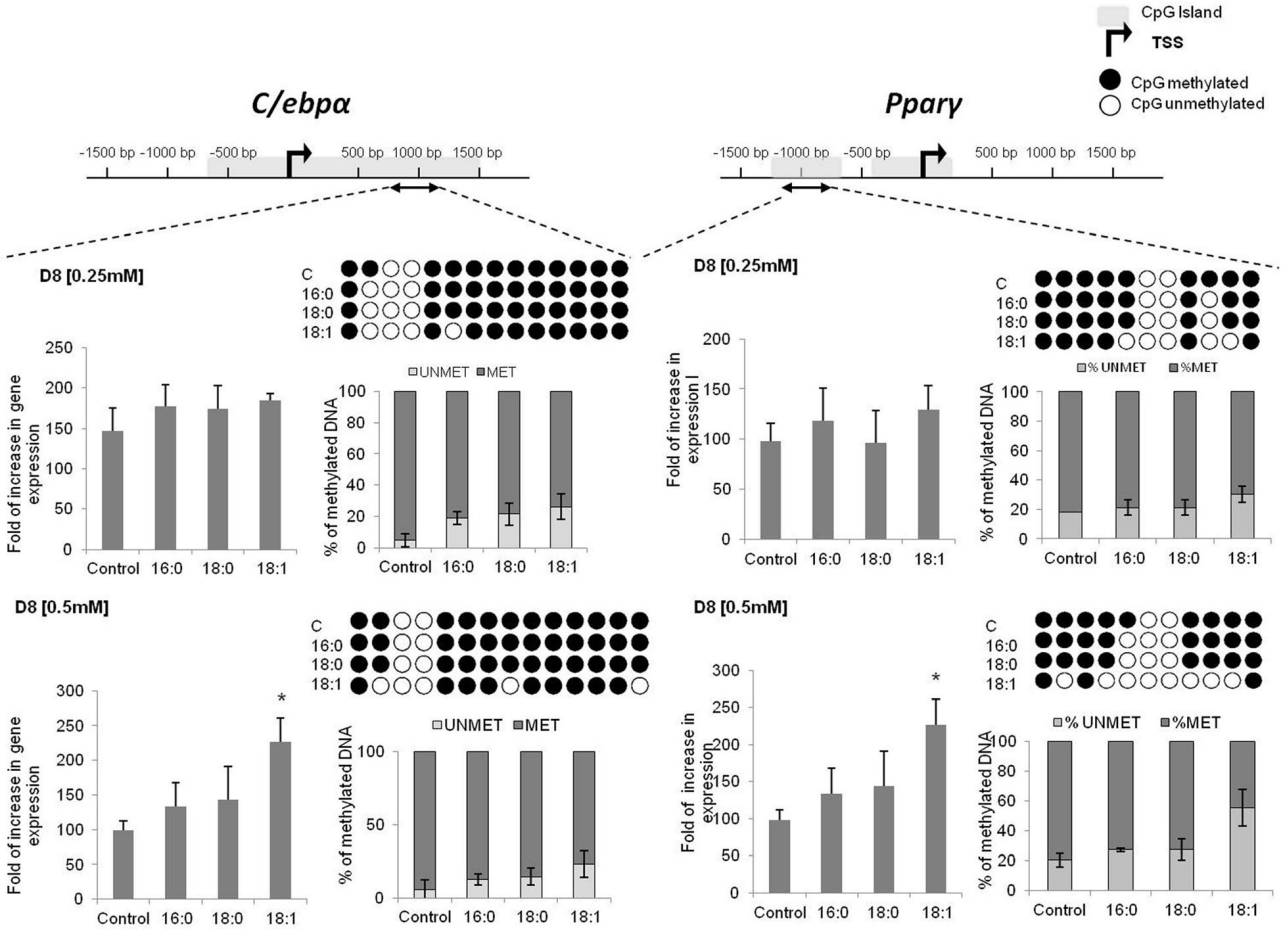
The expression level and methylation of promoters of late transcription factors in 3T3-L1 adipocytes. The expression and methylation study correspond to D8 of differentiation with two concentrations of fatty acid [0.25 mM and 0.5 mM]. A representative graph for each experimental group is presented along with the quantification data. The gene expression results are normalized to D0 (*β-actin* gene) and correspond to the rate of increase during differentiation. * p < 0.05

The promoter of *Pparγ* was methylated at a moderate rate within the investigated region in 3T3-L1. Comparing the rates of promoter methylation between cells, there were no statistically significant changes between experimental and control cells at D6 of differentiation, regardless of the concentration of fatty acids (data not shown). On the other hand, statistically important differences were noted in *Pparγ* promoter methylation between mature adipocytes, with lower methylation rates in cells differentiated with an excess of oleic acids [0.5 mM] compared to controls (18,1n-9, *p* = 0.0170, Fig. 5). In cells cultured with a lower concentration of fatty acids [0.25 mM], differences noted for *Pparγ* promoter methylation in cells treated with oleic acid did not reach significance, as compared to controls (*p* = 0.0518). No statistically significant changes in promoter methylation were detected in cells treated with palmitic and stearic acids, although the methylation rate was slightly decreased compared to control cells.

The methylation of the *C/ebpα* promoter decreased along with adipogenesis when cells were cultured with fatty acid concentrations of 0.5 mM, with a lower methylation rate at D8 of differentiation compared to D6 (data not shown). Similarly, there were no differences regarding the DNA methylation profile at D6 in the differentiation of *C/ebpα* promoter between experimental cells cultured with an excess of fatty acids [both concentrations] and controls. On the other hand, at D8 of adipogenesis, cells treated with 0.5 mM of oleic acid displayed significantly lower methylation profiles compared to controls (*p* = 0.0464). However, the mentioned effect was not observed in cells treated with 0.25 mM of fatty acid (Fig. 5).

These results suggest that the fatty acids, especially oleic acids may influence DNA methylation, especially at the promoter site of some genes, and that the influence operates in a dose-dependent manner.

### The effect of an excess of fatty acids on the phenotype of mature adipocytes (the end-point study) during adipogenesis

#### Insulin signaling and glucose metabolism

**Fig. 6:**
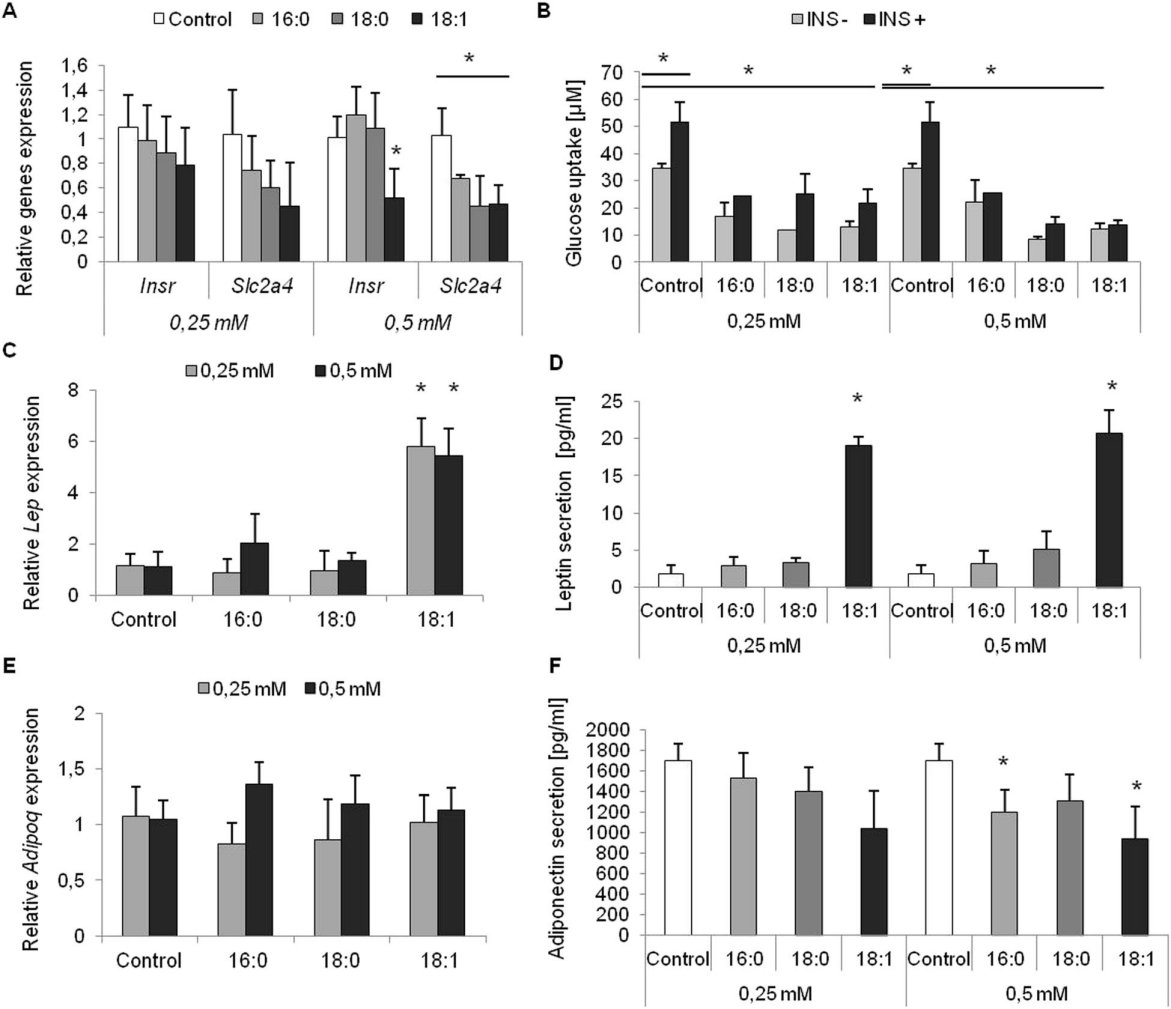
The phenotype of mature adipocytes differentiated with the excess of FFA. The phenotype effect of the excess of free fatty acids on insulin pathway expression genes (**a**), glucose uptake (**b**) and expression (**c**, **e**) and secretion (**d**, **f**) of main adipokines (leptin and adiponectin) in mature 3T3-L1 adipocytes. The gene expression presented as relative expression normalized to *β-actin* gene. * p < 0.05

In order to assess insulin signaling and glucose metabolism in adipocytes differentiated with an excess of FFA, we investigated expression rate of five genes belonging to the insulin signaling pathway: *Insr* (insulin receptor), *Pik3r1* (phosphoinositide-3-kinase regulatory subunit 1), *Ptpn1* (protein tyrosine phosphatase, non-receptor type 1), *Akt1* (thymoma viral proto-oncogene 1), and *Slc2a4* (solute carrier family 2 (facilitated glucose transporter), member 4). Two of five genes were differentially expressed in cells treated with fatty acids in a dose-dependent manner. The expression of *Insr* was slightly reduced in cells treated with oleic acid at a concentration of 0.25 mM, but in those treated with 0.5 mM of oleic acid, the reduction in expression was significant (*p* = 0.0251). The expression of *Slc2a4* was reduced in all experimental cells treated with fatty acids compared to control cells, with a greater reduction in cells cultured with 0.5 mM of FFA (16,0, *p* = 0.0194, 18,0, *p* = 0.0091, 18,1n-9, *p* = 0.0081). The reduction in *Slc2a4* in cells treated with 0.25 mM of fatty acids was considerable, however, and near the level of significance (Fig. 6 a).

These results suggest that glucose metabolism may be unsettled in 3T3-L1 adipocytes differentiated in an excess of fatty acids. Therefore, we performed a glucose uptake test to measure the glucose utilization in these cells at the basal stage and following insulin stimulation. The amount of glucose utilized by cells differentiated with fatty acids was significantly reduced comparing to control cells both at the basal stage (*p* = 0.0313) and following insulin stimulation (*p* = 0.0414). Glucose uptake in control cells increased following insulin stimulation (*p* = 0.0045). However, in cells cultured with fatty acids, the increase was moderate in adipocytes differentiated with fatty acids at a concentration of 0.25 mM (Fig. 6 b: 16:0, *p* = 0.1715; 18:0, *p* = 0.1187; 18:1n-9, *p* = 0.1667), and aborted in adipocytes differentiated with a concentration of 0.5 mM (Fig. 6 b: 16:0, *p* = 0.6194; 18:0,*p* = 0.0831; 18:1n-9, *p* = 0.4923). Similar effect of insulin resistance was observed for both saturated and monounsaturated fatty acids, as no stimulation of glucose uptake by insulin was observed or such stimulation was negligible.

In order to determine whether the divergences in insulin signaling genes expression might arise from a different promoter methylation profile, we measured the rates of promoter methylation using bisulfite sequencing. We observed no differences in promoter methylation patterns in the promoters of the *Slc2a4* and *Insr* genes between experimental and control cells.

#### Lipid metabolism

In order to assess lipid metabolism in 3T3-L1 cells differentiated with an excess of FFA (16:0, 18:0, 18:1n-9), we measured the expression of main lipogenic and lipolytic genes in these cells. The expression rate of three lipogenic genes, namely *Fasn* (fatty acid synthase), *Acaca* (acetyl-Coenzyme A carboxylase), and *Scd-1* (stearoyl-Coenzyme A desaturase 1), and of one lipolysis gene, *Lpl* (lipoprotein lipase), was investigated in experimental and control cells. There was no difference in gene expression between cells differentiated with an excess of FFA (for both concentrations) and control cells (data not shown).

A slight increase was noted in the expression rate of *Fasn* in cells treated with fatty acids at a concentration of 0.5 mM, compared to control 3 T3-L1; however, the changes in *Fasn* expression did not reach the level of significance. Despite gene expression, the total amount of accumulated fat was significantly higher in 3 T3-L1 adipocytes cultured with oleic acid (Fig. 2 b, p = 0.0000 for both fatty-acid concentrations) compared to control cells and adipocytes differentiated with an excess of either of the other two FFA.

Having observed no differences in gene expression rate, we did not expect to see a difference in the promoter methylation of lipid metabolism genes. Indeed, an initial study carried out on *Scd-1* and *Acaca* showed no differences in promoter methylation. Investigated fragments within promoters were totally unmethylated, with no differences between experimental and control cells.

#### Adipokine and cytokine expression and secretion

Subsequently, we became interested in the influence of an excess of free fatty acids on the expression rate and secretion of numerous adipokines and cytokines in 3T3-L1 mature adipocytes. Two adipokines, *Adipoq* (adiponectin), *Lep* (leptin), and three cytokines, pro-inflammatory *Il-6* (Interleukin 6) and *Tnfα* (Tumor necrosis factor, alpha) and anti-inflammatory *Il-10* (Interleukin 10), were analyzed in terms of their expression rate in mature 3T3-L1 adipocytes. There was no difference in the expression rates of the analyzed cytokines (data not shown). *Tnfα* exhibited a very low expression rate in adipocytes, both experimental cells and controls. Similarly, there were no differences in the expression rate of *Il-6* and *Il-10* between experimental cells and controls in terms of fatty-acid concentration (data not shown) Furthermore, there was no difference in IL-6 secretion by 3T3-L1 cells measured in the culture medium collected at the day 8 of differentiation.

On the other hand, we observed the influence of fatty acids, especially of oleic acid, on the expression rate of the *Lep* gene, which was expressed at a rate more than 5 times higher in adipocytes at both oleic acid concentrations (Fig. 6 c, p = 0.0051 and *p* = 0.0022 for 0.25 and 0.5 mM, respectively). This result was confirmed by measurement of Lep secretion using an ELISA kit in the cell medium collected at D8 of differentiation. The amount of secreted Lep was considerably greater in a cell medium collected from adipocytes treated with an excess of oleic acid (Fig. 6 d, p = 0.0089 and *p* = 0.0041 for 0.25 and 0.5 mM, respectively), with only a moderate increase in Lep secretion by cells cultured with two other fatty acids (palmitic and stearic) compared to control cells.

We also measured *Adipoq* gene expression and the secretion of adiponectin by experimental and control cells. There were no statistically important differences in the *Adipoq* expression rate in adipocytes differentiated with fatty acids compared to control cells. However, we noticed a reduction in the amount of adiponectin secreted by those cells, with the greatest reduction in cells cultured with an excess of oleic acid (Fig. 6 f, p= 0.0710 and* p* = 0.0010 for 0.25 and 0.5 mM, respectively), followed by palmitic acid (Fig. 6 f, p = 0.5970 and *p* = 0.0178 for 0.25 and 0.5 mM, respectively). The drop in adiponectin secretion was dose-dependent; adiponectin secretion decreased to a greater extent at the 0.5 mM concentration of FFA. The differences in adiponectin secretion by adipocytes cultured with an excess of stearic acid were not statistically significant at either concentration.

Despite the differences in the level of gene expression and adipokine secretion, we observed no changes in promoter methylation in *Lep* and *Adipoq* genes, which may suggest that adipokine expression and secretion are not regulated via epigenetic mechanisms.

## Discussion

In the present study, we tested the influence of the excess of three main fatty acids on adipogenesis based on the 3 T3-L1 cell model. These cells constitute the best established and most popular cell model for adipogenesis and for the study of metabolic disorders. In our study, we created a model of adipocyte formation with a constant excess of fatty acids delivered with food. We were interested in how an excess of fatty acids changes the differentiation process and phenotype of mature adipocytes. Based on our results, we observed that oleic acid (18:1n-9) enhances the induction of morphological changes and lipid accumulation in 3T3-L1 fibroblasts that appeared much earlier during cell differentiation than cells differentiated with the addition of saturated fatty acids and controls. On the other hand, the phenotype of mature adipocytes (especially insulin signaling pathway) was impaired in all experimental cells (differentiated with the excess of 16:0, 18:0 and 18:1n-9).

Adipogenesis is a complex process regulated by many factors, including epigenetics. Numerous data provide evidence on how epigenetics regulates the process of adipocytes formation, generally through DNA methylation and the introduction of various modifications in histones. Using knock-out approaches, numerous genes including *DNMT3a* [17]*, HDAC1* (histone deacetylase 1), and *HDAC2* (histone deacetylase 2) were proven to be crucial for proper adipogenesis [18, 19].

Some reports provide data indicating that diet (nutrition) may regulate adipogenesis by stimulating or repressing this process. The most widely studied diet is HFD; its influence on health as well as on the development of numerous metabolic diseases has been prolifically reported. We were interested how HFD influenced the process of adipogenesis and the phenotype of a mature adipocyte later in life. We also wished to evaluate which FFA (SFA or MUFA) exerted a greater influence on adipogenesis and phenotype of mature adipocytes. Therefore, 3T3-L1 fibroblasts were differentiated into mature adipocytes with an excess of fatty acids at two concentrations in order to assess the resulting influence on adipogenesis and phenotype of mature adipocytes in a dose-dependent manner. We observed that morphological changes as well that lipids accumulation appeared much earlier in cells treated with fatty acids, especially with oleic acid, resulting in a considerable increase in total accumulated fat at the end of adipogenesis. On the other hand, total lipid content was not significantly increased in cells differentiated with an excess of saturated fatty acids compared to control cells. The only observable difference was earlier visibility of lipid droplets, which appeared approximately on the third day of differentiation, generally 0.5–1 day earlier than in control cells. Previously, other researchers had published reports indicating that oleic acids might stimulate adipogenesis [12, 20], in which an increase in the expression rate of adipogenic genes was observed. Oleic acid promoted adipogenesis of hen preadipocytes into mature adipocytes by increasing the expression rates of mainly *Fabp4* and *C/ebpβ.* In findings similar to our results, other researchers showed that lipid droplets began to appear in cells cultured with oleic acid much earlier than in control cells, and that the total of lipids accumulated in mature adipocytes and other cells is significantly higher than in controls and in comparison to other examined fatty acids (16:0 and 18:0) [12, 15, 20, 21]. It is very likely that oleic acid stimulates lipid accumulation by promoting the expression rate of *Pparγ*. Previously, it had been found that overexpression of *Pparγ* stimulated lipid accumulation [22]; on the other hand, heterozygous *Pparγ*-deficient mice were characterized by lower lipid contents and smaller adipocytes [23]. In our study, late transcription factors were significantly increased in cells affected by oleic acid. The saturated fatty acids increased the expression of late transcription factor, similar to oleic acid, however, the increase was moderate and did not reach significance, as well as the total amount of accumulated lipids. Previously published results are contradictory regarding the effect of saturated fat on lipids accumulation. In bovine mammalian epithelial cells the increased lipids droplets were observed for unsaturated fatty acids, but not for saturated fats (palmitate and stearate) [21]. A similar effect was observed for 3T3-L1 cultured with palmitic acid [24]. On the other hand, Krautbauer et al. [25] showed higher triglyceride levels and the formation of larger lipid droplets in 3T3-L1 adipocytes cultured with both saturated and unsaturated fatty acids.

We showed that global DNA methylation was reduced in cells cultured with FFA, especially with oleic acids. The changes we observed in global DNA methylation during adipogenesis were similar to those observed in a previous study [26]. What is more, in that study, in cells with an excess of oleic acid (achieved by *Scd1* overexpression), global DNA methylation was lower, a finding similar to those in our study, proving that oleic acid influences overall global DNA methylation.

It had been shown previously that FFA might influence promoter methylation of numerous genes [26, 27]; thus we examined the promoter methylation profile of several genes that showed divergences in expression rates in experimental cells. In present paper, we showed that oleic acid influenced the promoter methylation of *Pparγ*. It is worth mentioning that the observed changes in *Pparγ* promoter methylation occurred in a dose-dependent manner. Some reports have indicated that the methylation of *Pparγ* promoter changes in metabolic disorders, especially type 2 diabetes [28, 29]. The demethylation of *Pparγ* promoter during adipocyte differentiation, as we have observed, is crucial for proper adipogenesis, and this had been reported previously [28]; however, data concerning *Pparγ* promoter in metabolic disorders are contradictory as well as being tissue-specific [28, 29]. For example, db/db mice were characterized by a lower level of *Pparγ* promoter methylation in subcutaneous adipose tissue, but by an increased level in visceral adipose tissue [28]; on the other hand, in humans with type 2 diabetes, methylation of *Pparγ* promoter was increased in terms of SAT compared to healthy subjects [29]. Thus it is difficult to correlate our results with the lower methylation rate of *Pparγ* promoter in cells cultured with FFA.

There is no data concerning *C/ebpα* expression and methylation in relation to metabolic disorders, especially adipogenesis. It is believed that *C/ebpα* is not as crucial factor for adipogenesis as *Pparγ*. There is no data concerning the influence of FFA on the expression of *C/ebpα*. We reported here for the first time that the methylation of *C/ebpα* promoter changed due to the influence of FFA, especially oleic acid, in a pattern similar to that observed for *Pparγ*. Generally, the methylation of *C/ebpα* decreased and the expression increased with an excess of oleic acid, however, observed only for the higher concentration of oleic acid [0.5 mM].

We also wished to assess the phenotype of mature adipocytes cultured with an excess of fatty acids throughout the process of adipogenesis. First, we checked insulin sensitivity by measuring the expression rate of insulin pathway genes and measuring glucose uptake by those cells. The *Insr* and *Slc2a4* genes encoding glucose transporter type 4 were downregulated in cells cultured with an excess of fatty acids, with the greatest drop in expression noted in cells cultured with oleic acid in a dose-dependent manner. The aberrant expression of *Insr* was selectively observed for oleic acid, with a greater decrease in a higher concentration of FA. The *Slc2a4* gene was downregulated by all three fatty acids; the decrease in the expression of *Slc2a4* negatively correlated with the concentration of fatty acids. What is more, the lowest level of expression was noted for oleic acid. Similar results had been obtained previously [15], where moderate decreases in expression in cells treated with palmitic and stearic acids were observed, but a significant drop in *Slc2a4* expression was seen in cells treated with oleic acid. Moreover, we have shown that the effect of FFA on *Slc2a4* expression occurs in a dose-dependent manner.

These results correspond to glucose uptake tests conducted on control and experimental cells in which 0.25 mM of fatty acids reduced the response of cells to insulin to a small extent. In some cases, insulin increased the total glucose uptake in cells differentiated with an excess of fatty acid. When cells were differentiated with a concentration of fatty acids of 0.5 mM, only cells treated with stearic acid preserved the respond for insulin. In cells treated with palmitic and oleic acids, insulin sensitivity was abolished. Nevertheless, the total amount of glucose uptake was much lower in experimental cells than in control cells at both basal and insulin-stimulated stages. Similar results obtained Xie and co [20], which also displayed a decrease Glut4 level in 3T3-L1 differentiated with oleic acid and impaired phosphorylation of Akt, suggesting resistance to insulin in those cells.

These results contradict those of another study [30] in which oleic acids were shown to stimulate glucose uptake; however, the concentration of oleic acid used in that study was much lower than that used in our study (0.1 as opposed to 0.5 mM). Moreover, further research on aberrant insulin sensitivity in cells differentiated with an excess of oleic acid (especially with a concentration of 0.5 mM) yielded results indicating a lower level of adiponectin secretion. Adiponectin in adipocytes, apart from its many other roles, increases insulin-stimulated glucose uptake [31] and has been shown to be reduced in insulin-resistant adipocytes [32]. The expression of the *Adipoq* gene in experimental cells was not affected by fatty acids; on the other hand, the secretion of adiponectin was significantly lower in cells treated with 0.5 mM of fatty acid, especially palmitic and oleic acid. A previously published report provided data indicating that a lower serum adiponectin level was correlated with a higher prevalence of metabolic syndrome, including insulin resistance, and was considered a strong risk factor in the development of insulin resistance [33]. In contradiction to another study [32], the methylation profile of the promoter of the *Adipoq* gene was not changed in experimental cells despite a lower adiponectin secretion rate and an aberrant response in cells treated with FFA following insulin stimulation. Based on Kim et al., methylation of *Adipoq* promoter should be differential in insulin-resistant cells [32]. Additional studies must be performed in order to elucidate this issue.

During our investigations, we observed a considerable increase in *Lep* expression and secretion in cells cultured with an excess of oleic acid compared to controls and cells cultured with saturated fats. Generally, leptin is considered an energy sensor and overfeeding signal in adipocytes; the higher the energy reserve, the higher the level of *Lep* expression [34]. Based on our results, we were able to conclude that 3T3-L1 cells differentiated with oleic acid were more “obese” than control cells or 3T3-L1 cells cultured with SFA. The overexpression of the *Lep* gene following treatment with oleic acid had been reported previously [12, 15, 21]; however, no researchers reported more than a fivefold increase in *Lep* expression and secretion. Yonezawa et al. reported a 1.4-fold increase in leptin mRNA in bovine mammary epithelial cells following oleic acid treatment [21]; however, no changes in leptin mRNA level were observed in cells treated with palmitic and stearic acids. Likewise, we observed no significant changes in *Lep* expression in cells treated with saturated fats. However, some data show a reduction in expression of the *Lep* gene in adipocytes treated with oleic acids [2], which contradicts our results. Overall, including the amount of accumulated fat, and accounting for the fact that leptin expression correlates with the size of the adipocytes, our results appear consistent.

It has been shown by numerous investigators [35, 36] that methylation of the promoter of the *Lep* gene is affected by HFD, which has an effect on the increased expression of *Lep* in obesity and obesity-related disorders. We observed no changes in *Lep* promoter methylation caused by an excess of SFA or MUFA; this finding was similar to those of others who observed no changes in *Lep* promoter methylation rate due to dietary factors [37].

## Conclusions

To summarize, oleic acids enhanced the changes in morphology and lipids accumulation in 3T3-L1 preadipocytes during the differentiation process. Furthermore, oleic acid affected the methylation of *C/ebpα* and *Pparγ* promoters and increasing their expression in a dose-dependent manner. Moreover, free fatty acids influenced the phenotype of mature adipocytes cultured in vitro, especially the insulin-signaling pathway and adipokine secretion, by impairing the glucose uptake process and causing overall changes similar to insulin-resistant cells.

## Data Availability

The datasets used and/or analyzed during the current study will be available from the corresponding author on reasonable request.

## Abbreviations

16:0: Palmitic acid
18:0: Stearic acid
18:1n-9: Oleic acid
BSA: Bovine serum albumin
CpG: Cytosine-phosphate-Guanine
DMSO: Dimethyl Sulfoxide
DNA: Deoxyribonucleic acid
FFA: Free fatty acids
HFD: High-fat diet
IBMX: 3-Isobutyl-1-methylxanthine
MCE: Mitotic clonal expansion
MSC: Mesenchymal stromal cells;
MTT: 3-(4,5-dimethylthiazol-2-yl)-2,5-diphenyltetrazolium bromide, a tetrazole
MUFA: monounsaturated fatty acid
PBS: Phosphate Buffer Solution
PFA: Paraformaldehyde
RNA: Ribonucleic acid
SFA: Saturated fatty acid

## References

[1] Siersbaek R, Mandrup S (2011). Transcriptional Networks Controlling Adipocyte Differentiation. Cold Spring Harb Symp Quant Biol.

[2] Hwang CS, Loftus TM, Mandrup S, Lane MD (1997). Adipocyte differentiation and leptin expression. Annu Rev Cell Dev Biol.

[3] Ali AT, Hochfeld WE, Myburgh R, Pepper MS (2013). Adipocyte and adipogenesis. Eur J Cell Biol.

[4] Musri MM, Párrizas M (2012). Epigenetic regulation of adipogenesis. Curr Opin Clin Nutr Metab Care.

[5] Li Hong-xing, Xiao Lei, Wang Cheng, Gao Jia-li, Zhai Yong-gong (2010). Epigenetic regulation of adipocyte differentiation and adipogenesis. Journal of Zhejiang University SCIENCE B.

[6] Deaton AM, Bird A (2011). CpG islands and the regulation of transcription. Genes Dev.

[7] Deans C, Maggert KA (2015). What Do You Mean, “Epigenetic”?. Genetics.

[8] Jiménez-Chillarón JC, Díaz R, Martínez D, Pentinat T, Ramón-Krauel M, Ribó S (2012). The role of nutrition on epigenetic modifications and their implications on health. Biochimie.

[9] Jacobsen SC, Brøns C, Bork-Jensen J, Ribel-Madsen R, Yang B, Lara E (2012). Effects of short-term high-fat overfeeding on genome-wide DNA methylation in the skeletal muscle of healthy young men. Diabetologia.

[10] Malmgren Siri, Spégel Peter, Danielsson Anders P.H., Nagorny Cecilia L., Andersson Lotta E., Nitert Marloes Dekker, Ridderstråle Martin, Mulder Hindrik, Ling Charlotte (2013). Coordinate Changes in Histone Modifications, mRNA Levels, and Metabolite Profiles in Clonal INS-1 832/13 β-Cells Accompany Functional Adaptations to Lipotoxicity. Journal of Biological Chemistry.

[11] García-Escobar E, Monastero R, García-Serrano S, Gómez-Zumaquero JM, Lago-Sampedro A, Rubio-Martín E (2017). Dietary fatty acids modulate adipocyte TNFa production via regulation of its DNA promoter methylation levels. J Nutr Biochem.

[12] Regassa A, Kim WK (2013). Effects of oleic acid and chicken serum on the expression of adipogenic transcription factors and adipogenic differentiation in hen preadipocytes. Cell Biol Int.

[13] Liao K, Yan J, Mai K, Ai Q (2015). Dietary olive and Perilla oils affect liver mitochondrial DNA methylation in large yellow croakers. J Nutr.

[14] Zhu L, Shi C, Ji C, Xu G, Chen L, Yang L (2013). FFAs and adipokine-mediated regulation of hsa-miR-143 expression in human adipocytes. Mol Biol Rep.

[15] Yanting C, Yang QY, Ma GL, Du M, Harrison JH, Block E (2018). Dose- and type-dependent effects of long-chain fatty acids on adipogenesis and lipogenesis of bovine adipocytes. J Dairy Sci.

[16] Kumaki Y, Oda M, Okano M (2008). QUMA: quantification tool for methylation analysis. Nucleic Acids Res.

[17] You D, Nilsson E, Tenen DE, Lyubetskaya A, Lo JC, Jiang R, et al. Dnmt3a is an epigenetic mediator of adipose insulin resistance. Elife. 2017;6. 10.7554/eLife.30766 PMC5730374 29091029

[18] Zhang Q, Ramlee MK, Brunmeir R, Villanueva CJ, Halperin D, Xu F (2012). Dynamic and distinct histone modifications modulate the expression of key adipogenesis regulatory genes. Cell Cycle.

[19] Ge K (2012). Epigenetic regulation of adipogenesis by histone methylation. Biochimica et Biophysica Acta (BBA) Gene Regulatory Mechanisms.

[20] Xie W, Hamilton JA, Kirkland JL, Corkey BE, Guo W (2006). Oleate-induced formation of fat cells with impaired insulin sensivitity. Lipids.

[21] Yonezawa T, Yonekura S, Kobayashi Y, Hagino A, Katoh K, Obara Y (2004). Effects of long-chain fatty acids on cytosolic triacylglycerol accumulation and lipid droplet formation in primary cultured bovine mammary epithelial cells. J Dairy Sci.

[22] Tontonoz P, Hu E, Spiegelman BM (1994). Stimulation of adipogenesis in fibroblasts by PPAR gamma 2, a lipid-activated transcription factor. Cell..

[23] Kubota N, Terauchi Y, Miki H, Tamemoto H, Yamauchi T, Komeda K (1999). PPAR gamma mediates high-fat diet-induced adipocyte hypertrophy and insulin resistance. Mol Cell.

[24] Takahashi K, Yamaguchi S, Shimoyama T, Seki H, Miyokawa K, Katsuta H (2008). JNK- and IκB-dependent pathways regulate MCP-1 but not adiponectin release from artificially hypertrophied 3T3-L1 adipocytes preloaded with palmitate in vitro. Am J Physiol-Endocrinol Metab.

[25] Krautbauer S, Eisinger K, Neumeier M, Hader Y, Buettner R, Schmid PM, et al. Free Fatty Acids, Lipopolysaccharide and IL-1α Induce Adipocyte Manganese Superoxide Dismutase Which Is Increased in Visceral Adipose Tissues of Obese Rodents. Lluch GL, editor. PLoS ONE 2014, 9:e86866. Available from: https://dx.plos.org/10.1371/journal.pone.0086866 10.1371/journal.pone.0086866 PMC3901719 24475187

[26] Malodobra-Mazur M, Dziewulska A, Kozinski K, Dobrzyn P, Kolczynska K, Janikiewicz J (2014). Stearoyl-CoA desaturase regulates inflammatory gene expression by changing DNA methylation level in 3T3 adipocytes. Int J Biochem Cell Biol.

[27] Perfilyev A, Dahlman I, Gillberg L, Rosqvist F, Iggman D, Volkov P (2017). Impact of polyunsaturated and saturated fat overfeeding on the DNA-methylation pattern in human adipose tissue: a randomized controlled trial. Am J Clin Nutr.

[28] Fujiki Katsunori, Kano Fumi, Shiota Kunio, Murata Masayuki (2009). Expression of the peroxisome proliferator activated receptor gamma gene is repressed by DNA methylation in visceral adipose tissue of mouse models of diabetes. BMC Biology.

[29] Nilsson E, Jansson PA, Perfilyev A, Volkov P, Pedersen M, Svensson MK (2014). Altered DNA methylation and differential expression of genes influencing metabolism and inflammation in adipose tissue from subjects with type 2 diabetes. Diabetes..

[30] Tsuchiya A, Nagaya H, Kanno T, Nishizaki T (2014). Oleic acid stimulates glucose uptake into adipocytes by enhancing insulin receptor signaling. J Pharmacol Sci.

[31] Ruan H, Dong LQ (2016). Adiponectin signaling and function in insulin target tissues. J Mol Cell Biol.

[32] Kim AY, Park YJ, Pan X, Shin KC, Kwak S-H, Bassas AF (2015). Obesity-induced DNA hypermethylation of the adiponectin gene mediates insulin resistance. Nat Commun.

[33] Cho S-A, Joo HJ, Cho J-Y, Lee SH, Park JH, Hong SJ (2017). Visceral fat area and serum Adiponectin level predict the development of metabolic syndrome in a community-based asymptomatic population. PLoS One.

[34] Pandit R, Beerens S (2017). Adan R a. H. Role of leptin in energy expenditure: the hypothalamic perspective. Am J Physiol Regul Integr Comp Physiol.

[35] García-Cardona MC, Huang F, García-Vivas JM, López-Camarillo C, del Río Navarro BE, Navarro Olivos E (2014). DNA methylation of leptin and adiponectin promoters in children is reduced by the combined presence of obesity and insulin resistance. International J Obesity.

[36] Zwamborn RAJ, Slieker RC, Mulder PCA, Zoetemelk I, Verschuren L, Suchiman HED (2017). Prolonged high-fat diet induces gradual and fat depot-specific DNA methylation changes in adult mice. Sci Rep.

[37] Fan C, Liu X, Shen W, Deckelbaum RJ, Qi K (2011). The Regulation of Leptin, Leptin Receptor and Pro-opiomelanocortin Expression by N-3 PUFAs in Diet-Induced Obese Mice Is Not Related to the Methylation of Their Promoters. Nutr Metab (Lond).

